# Extensive Lesions in Rat Insular Cortex Significantly Disrupt Taste Sensitivity to NaCl and KCl and Slow Salt Discrimination Learning

**DOI:** 10.1371/journal.pone.0117515

**Published:** 2015-02-06

**Authors:** Ginger D. Blonde, Michelle B. Bales, Alan C. Spector

**Affiliations:** Department of Psychology and Program in Neuroscience, Florida State University, Tallahassee, FL, United States of America; Duke University Medical Center, UNITED STATES

## Abstract

While studies of the gustatory cortex (GC) mostly focus on its role in taste aversion learning and memory, the necessity of GC for other fundamental taste-guided behaviors remains largely untested. Here, rats with either excitotoxic lesions targeting GC (n = 26) or sham lesions (n = 14) were assessed for postsurgical retention of a presurgically LiCl-induced conditioned taste aversion (CTA) to 0.1M sucrose using a brief-access taste generalization test in a gustometer. The same animals were then trained in a two-response operant taste detection task and psychophysically tested for their salt (NaCl or KCl) sensitivity. Next, the rats were trained and tested in a NaCl vs. KCl taste discrimination task with concentrations varied. Rats meeting our histological inclusion criterion had large lesions (resulting in a group averaging 80% damage to GC and involving surrounding regions) and showed impaired postsurgical expression of the presurgical CTA (LiCl-injected, n = 9), demonstrated rightward shifts in the NaCl (0.54 log_10_ shift) and KCl (0.35 log_10_ shift) psychometric functions, and displayed retarded salt discrimination acquisition (n = 18), but eventually learned and performed the discrimination comparable to sham-operated animals. Interestingly, the degree of deficit between tasks correlated only modestly, if at all, suggesting that idiosyncratic differences in insular cortex lesion topography were the root of the individual differences in the behavioral effects demonstrated here. This latter finding hints at some degree of interanimal variation in the functional topography of insular cortex. Overall, GC appears to be necessary to maintain normal taste sensitivity to NaCl and KCl and for salt discrimination learning. However, higher salt concentrations can be detected and discriminated by rats with extensive damage to GC suggesting that the other resources of the gustatory system are sufficient to maintain partial competence in these tasks, supporting the view that such basic sensory-discriminative taste functions involve distributed processes among central gustatory structures.

## Introduction

The gustatory cortex (GC) is conventionally defined as a zone of agranular/dysgranular insular cortex flanking the middle cerebral artery containing taste-responsive neurons [1–7], and has been a focus for studying taste aversion learning and memory in the rat. Bilateral lesions to GC are reported to virtually eliminate the retention of a presurgically conditioned taste aversion (CTA), in which animals are trained to avoid a tastant by pairing its ingestion with visceral malaise [2; 8–12]. Similarly, postsurgical acquisition is often, but not always, impaired [8–9,11,13–21]. The effects of pharmacological manipulations during CTA induction also suggest involvement of GC in taste memory consolidation [22–25]. However, recent findings from our laboratory, while confirming that insular cortex lesions compromise CTA learning, question whether the critical site encompasses GC alone or rather also involves adjacent sites implicated in visceroception [26]. Interestingly, intake tests suggest that unconditioned preference and avoidance appear normal under most, but not all, test conditions after GC damage [9, 27–29]. In agreement, extensive bilateral damage to GC has little effect on unconditioned concentration-dependent responsiveness to sucrose or quinine [30].

Collectively, these prior studies suggest that GC is unnecessary for normal unconditioned hedonic responsiveness to tastants and that its role in taste aversion learning may be in conjunction with visceroceptive areas of insular cortex. This raises the question as to the functional role of GC. An early hypothesis by Pfaffmann and colleagues (1977) postulated that the thalamocortical gustatory pathway provides information regarding stimulus identification and discriminability, while the divergent ventral forebrain gustatory pathway is involved in taste-guided motivation and affect [31]. However, despite evidence suggesting that humans experience hypogeusia and difficulty identifying tastes after damage to insular cortex [32–35], whether such lesions alter detection of or discrimination between taste stimuli remains untested psychophysically in the rat – the model of choice for the experimental analysis of the functional neurocircuitry of the gustatory system.

Virtually all lesion studies targeting GC have depended on tasks assessing conditioned or unconditioned palatability and most have involved the use of intake tests which can be influenced by nontaste factors. Here, we applied an operant two-response taste discrimination task to assess the ability of rats with large bilateral GC lesions to detect and discriminate two common salts irrespective of their inherent palatability. The task that we used has some distinct benefits in the assessment of sensory-discriminative taste function. First, stimulus samples are very small and immediate responses are measured. These features increase the confidence that the responses are based on the orosensory properties of the stimulus. Second, taste serves as a discriminative cue guiding responses to the proper water source. As such, the responses are motivated by the water-deprived animal’s drive to rehydrate and not the hedonic characteristics of the stimulus [36]. Because the insular cortex is implicated in CTA retention we also presurgically conditioned rats to avoid sucrose and tested retention and specificity of the CTA postsurgically in a brief-access taste test.

## Materials and Methods

### Subjects

Forty-five male Sprague-Dawley rats weighing 300–350g at the start of the experiment were used as subjects. Animals were individually housed in polycarbonate cages in a room with a 12-hr light/dark cycle; temperature and humidity were automatically controlled. Rats had *ad libitum* access to standard laboratory chow (Rodent Diet 5001; PMI) throughout the experiment. Access to reverse-osmosis deionized water (DW) was contingent upon training and testing phases (see below for descriptions, Table 1 for summary). Procedures were approved by the Animal Care and Use Committee at Florida State University (protocol 1012). Surgery was performed under isoflurane anesthesia with the delivery of postsurgical analgesics, and all efforts were made to minimize suffering.

**Table 1:** Schedule of experimental phases.

Phase	Days	Stimuli	Stimulus Presentation
Conditioned Taste Aversion (CTA)^1^			
Acclimation	4	Water	*Ad libitum*
Conditioning Trial^2^	1	0.1 M Sucrose (CS)	*Ad libitum*
**Surgery / Recovery**			
CTA Retention			
Water Training^3^	5	Water	Semirandom^5^
Retention Test^3^	1	CS and prototypical tastants	Semirandom
2-bottle Preference	2	CS vs. water	*Ad libitum*
NaCl Detection^4^			
Stationary Training	3	Water	*Ad libitum*
Side Training	4	0.4 M NaCl or water	Constant
Alternation	4	0.4 M NaCl and water	Semirandom
Random Training I	2	0.4 M NaCl and water	Semirandom
Random Training II	2	0.4 M NaCl and water	Semirandom
Testing	28	NaCl and water	Semirandom
KCl Detection^4^			
Side Training	2	0.4 M KCl or water	Constant
Random Training	7	KCl and water	Semirandom
Testing	22	KCl and water	Semirandom
Salt Discrimination^4^			
Side Training	4	0.4 M NaCl or 0.6 M KCl	Constant
Random Training I	10	0.4 M NaCl and 0.6 M KCl	Semirandom
Random Training II	4	NaCl and KCl	Semirandom
Testing	6	NaCl and KCl	Semirandom

^1^Access to one stimulus for 15 minutes in the home cage. Water was given for 30-min, ∼5-hr after completion of the morning session.

^2^The unconditioned stimulus immediately followed CS presentation: 0.15 M LiCl or NaCl (2 mEq/kg, i.p.)

^3^Brief-access trials (10-s duration) in 30-min sessions in the gustometer

^4^Two-response operant task in 30-min sessions in the gustometer.

^5^Stimuli presented in randomized blocks with a stimulus presentation probability of 0.5.

### Stimuli

All stimuli were made daily using reagent-grade chemicals dissolved in DW. For the CTA, the conditioned stimulus (CS) used was 0.1 M sucrose (Mallinkrodt Baker, Inc.). The unconditioned stimulus (US) was 0.15 M LiCl or NaCl (Sigma Aldrich; Fisher Scientific, respectively). For the postsurgical CTA retention test, the stimuli were: 0.03, 0.1, and 0.3 M sucrose, 0.2 M NaCl, 0.1 mM quinine hydrochloride (Sigma Aldrich), 10 mM citric acid (Sigma Aldrich), and DW. The NaCl and KCl (Fisher Scientific) concentrations used in detection threshold training and/or testing ranged from 0.0004 M to 0.4 M and 0.0025 M to 0.8 M, respectively. During the salt discrimination task, rats were trained and/or tested with concentrations ranging from 0.003 to 0.4 M NaCl and 0.04 to 0.6 M KCl.

### Apparatus

In some of the procedures, training and testing was conducted in a specialized stimulus delivery device called a *gustometer*. The design of this apparatus was based on that used for testing mice [37] but modified for use with rats. The testing chamber consists of a rectangular plastic cage (35.5 cm x 24 cm x 24 cm) with a wire mesh floor and a stainless steel front panel with three access slots (0.75 cm x 2.5 cm) set 3.8 cm above the cage floor. Taste samples are available via a borosilicate glass ball (1.4 cm diameter) which spins around a horizontal axis behind the central access slot. Licks are registered by a force transducer connected to the sample ball; this precludes the need to pass electrical current through the animal which is the case for many conventional lick circuits. Fluid is deposited onto the sample ball via Teflon tubing (ID 0.13 cm, OD 0.19 cm) connected to syringes mounted to a pump that advances the plunger via a stepping motor. Once two licks occur within 250-ms, a preload of ∼10 μl of the sample fluid is deposited to coat the sample ball. Then, the sample is delivered onto the ball upon each subsequent lick in ∼5 μl increments. During the intertrial interval, the sample ball is retracted into a washer where it is rinsed with DW and dried with pressurized air before returning to its position in front of the access slot in preparation for a new trial. A fixed reinforcement ball (1.3 cm diameter, polyoxymethylene) is stationed behind each access slot to either side of the sample ball. Each ball registers licks as described for the sample ball, but fluid is delivered by the pumps via PTFE tubing connected to a stainless-steel tube threaded through the ball. When necessary, access to the reinforcement balls can be manually blocked by stainless steel shutters. The cage and fluid delivery system are housed within a sound-attenuating chamber and background noise is presented during the session, both of which were designed to reduce extraneous auditory signals. A stainless steel shield blocks everything except the sample ball from the view of the rat to limit potential visual cues. Lastly, air is drawn away from the sample ball via a plastic duct connected to an exhaust fan mounted in the wall of the sound attenuation chamber to help limit extraneous olfactory cues.

### Surgery

Six days after presurgical conditioning (see below), the rats were divided into surgical groups that received either bilateral excitotoxic lesions to the gustatory cortex (GCX, n = 31) or sham surgery (SHAM, n = 14) with approximately half assigned to each unconditioned stimulus (US) group, balanced by body mass, conditioned stimulus (CS) intake, and which apparatus they were to be assigned during postsurgical testing. More animals were placed in the GCX groups than in the SHAM groups in anticipation of some incomplete lesions.

The GCX rats were anesthetized with isoflurane in an induction chamber (5%) and mounted on a stereotaxic apparatus using nontraumatic ear bars with isoflurane administered via a nosecone (2.0–2.5%) for the duration of surgery. A midline incision exposed the skull, which was leveled between bregma and lambda and between right and left at +3.0 mm lateral to bregma. Holes were drilled through the skull bilaterally to expose dura over the injection targets. Because the GC traverses ∼2.5 mm along the anterior-posterior (A/P) axis, two injections were made in each hemisphere, one rostral (+1.4 mm A/P, +5.2 mm M/L, -6.4 mm D/V, relative to bregma) and one caudal (+0.5 mm A/P, +5.6 mm M/L, -6.6 mm D/V, relative to bregma). A pulled glass pipette (tip diameter: ∼50 μm) was fitted to the end of the barrel of a 1.0 μl Hamilton syringe and sealed with paraffin. The syringe was filled with 20 mg/ml ibotenic acid (dissolved in PBS vehicle). For each injection, the pipette was lowered into position and left in place for 5-min prior to and after a 180 nl infusion of ibotenic acid that was pressure-injected over 15-min. If, after the pipette was removed, ibotenic acid could not be expelled, the pipette was replaced and the infusion was repeated. The incision was closed with wound clips. SHAM rats received an identical surgery, except that the infusion was of the PBS vehicle. On the day of and for three days after surgery, each rat received an injection of Procaine G penicillin (30,000 units, sc) and ketorolac tromethamine (2 mg/kg body mass, sc). Wound clips were removed 7–10 days following surgery.

### CTA Training and Testing

**Presurgical Conditioning**. Prior to the start of conditioning, water bottles were removed from the home cage. The following morning, rats received a single bottle of DW for 15-min. Approximately five hours later, rats were given free access to water for 30-min. This schedule continued until all rats had consumed fluid during the morning intake test for at least 3 days, which occurred after 4 days of training. The following day, animals received 0.1 M sucrose (CS) for 15-min during the morning intake session, immediately after which they were given an injection of either 0.15 M LiCl or NaCl (US; 2 mEq/kg, i.p.). All rats received water during the 30-min afternoon access period, and then water bottles were returned for rehydration. Injection groups were balanced on the basis of body mass and DW consumed during the last 15-min access training period.

**Postsurgical CTA Testing in Gustometer**. Two weeks after the last surgery, water bottles were removed from the home cages. Rats were placed in the gustometer and were trained and tested in a brief-access paradigm while water-deprived in 30-min sessions. Fluid was only available to the animal during the session. The reinforcement balls in the gustometer, being unnecessary for this test, were blocked by shutters. For the first two days of training, the sample ball was stationary and DW was available upon each lick throughout the session. On the following four days of training, 10-s water trials were delivered from seven syringes in randomized blocks of seven trials. A trial began once the rat licked the sample ball twice within 250-ms, and during the trial the rat could take as many licks as possible. Each stimulus trial was preceded by a 5-lick (in a maximum of 2-s) DW rinse. Between each rinse and stimulus trial was a 6-s intertrial interval, during which the stimulus ball was retracted and rinsed with DW, dried with pressurized air, and returned to position for the next trial. On the day following the last training session, a single CTA retention test session was conducted. This test was similar to the prior session except that instead of water, the test stimuli were delivered in randomized blocks of seven trials. The use of this paradigm allows the assessment of CTA retention along with generalization to other concentrations and/or stimuli via the measurement of licking responses to the stimulus array, including the CS, in short duration trials that minimize postingestive feedback, therefore increasing confidence that the behavior is guided by taste [36]. Following the test, water bottles were returned to the animals to allow them to rehydrate.

**Two-bottle Preference Testing of CTA**. On the day following the CTA retention brief-access test, a 48-h two-bottle preference test began wherein each rat, in its home cage, was presented with one bottle filled with DW and a second bottle filled with 0.1 M sucrose. After 24 h, both bottles were rinsed and refilled and their positions on the cage switched to control for potential side bias. Intake (in ml) was measured at 24 and 48 h. The use of this test allows the measurement of preference for the CS relative to water, and is the most common method used in studying the role of GC in CTA retention and acquisition.

### Operant Training and Psychophysical Testing of Salt Taste Sensitivity

Detection for both NaCl and KCl was conducted using a two-response operant task, with a modified method of limits procedure for stimulus presentation based on that described previously [38]. This task requires the thirsty animal to sample a small volume of stimulus and quickly determine how to respond based on which stimulus (e.g., NaCl or water) is presented in order to receive a water reward. Across test sessions, the concentration of the test stimulus is decreased until the animals can no longer discriminate the taste solution from water. Water bottles were removed from cages to begin training in the gustometer described above. Throughout all remaining phases of training and testing, water bottles were removed from the cages the afternoon before testing began for the week and were returned after completion of the final session of the week. Water was acquired via stimulus sampling and reinforcement for correct responses in the task. Sessions lasted 30 minutes.

**Fig 1:**
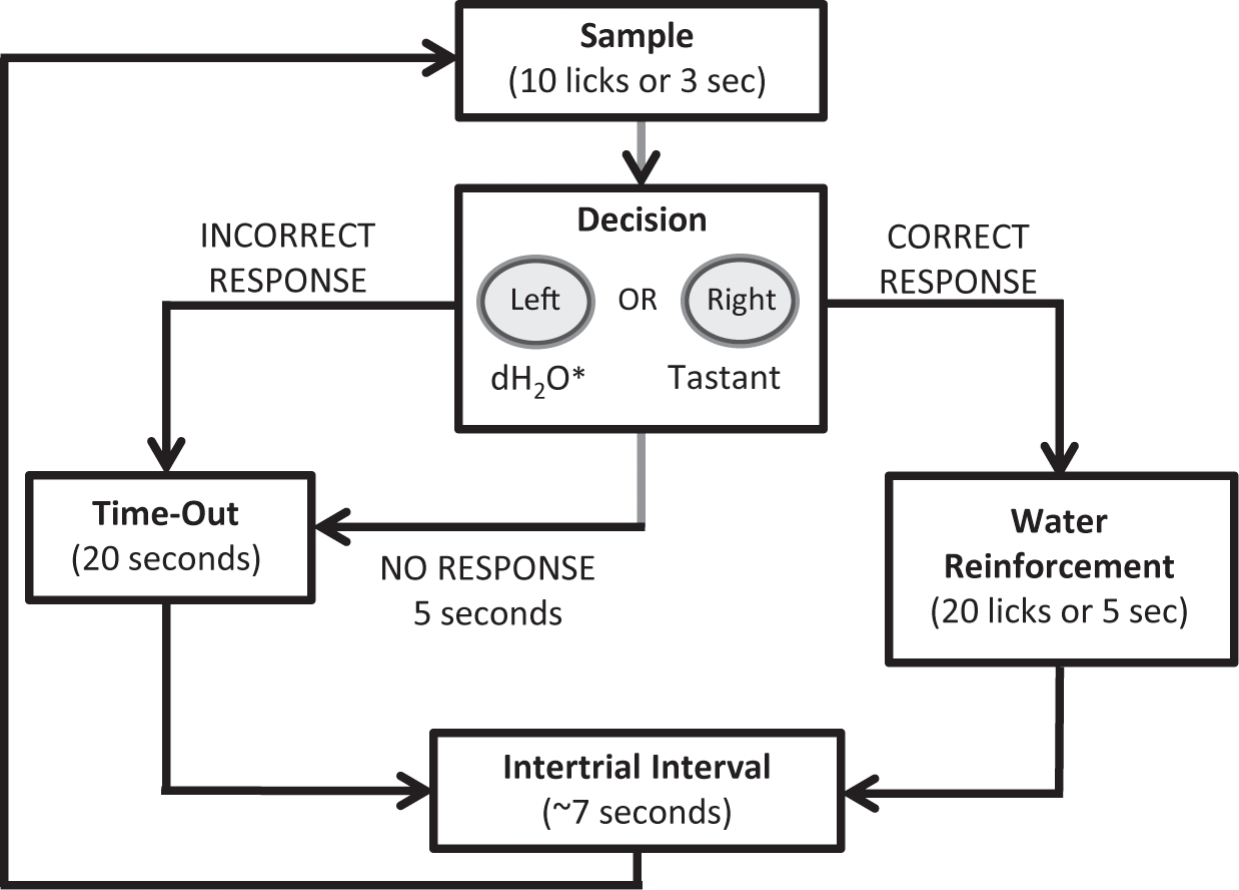
Trial Structure of operant taste discrimination task. Each trial began when the rat licked twice within 250 ms. See text for more details.

**Trial Structure**. At the beginning of the session, the sample ball was washed and then positioned in front of its access slot. The rat initiated a trial by licking the dry sample ball twice within 250-ms, which ensured that the rat was actively engaged in licking. During the sample phase (see Fig. 1), rats could receive up to 10 licks within 3-s, after which the sample ball was retracted. The rat then had a short duration to respond on one of the two reinforcement balls (limited hold). Correct responses were reinforced with up to 20 licks of DW (within 5-s), whereas incorrect or no responses resulted in a time-out with no water reinforcement. The duration of the limited hold (time to respond) varied during training and is specified in the descriptions of phases below. A ∼7-s intertrial interval then commenced, during which the sample ball was cleaned as described above in preparation for a new trial.

**NaCl Detection**. In this experimental phase, animals were trained and then tested in a two-response operant task that required the animals to respond one way if the stimulus presented was a NaCl concentration and another way if the stimulus presented was DW. By successively lowering NaCl concentration across sessions and requiring the animal to detect the presence of NaCl, it is possible to determine each animal’s detection threshold for NaCl (specifically, the EC_50_ value from a logistic function fit based on the individual animal’s performance), a measure of its sensitivity to the stimulus [36].

Stationary Training familiarized the animal with the stimulus and reinforcement balls from which it could receive fluid. For one day each, water was available *ad libitum* from the sample, left reinforcement, and right reinforcement, in that order. Shutters blocked access to the other potential sources of fluid.

For Side Training, the taste sample solutions were first paired with one of the reinforcement spouts. During each of 4 sessions rats received either 0.4 M NaCl or water from the stimulus ball, and they had access to only the reinforcement ball assigned to that stimulus, with the other blocked via shutter. Spout assignments were counterbalanced, with approximately half of each group having NaCl assigned to the left reinforcement spout, and with the other half having NaCl assigned to the right reinforcement spout. The limited hold was 180 s. Upon a correct response, the rat received 20 licks of water (within 5 s) from the reinforcement ball. If the rat did not respond by the end of the limited hold, there was no time-out, but the rat did not receive a water reinforcer.

The next phase of training was Alternation, wherein rats received both stimuli during the same session and had to respond correctly on the associated reinforcement ball to receive fluid. The same stimulus was presented in subsequent trials until the rat reached a criterion of nonconsecutive correct responses, which decreased across 4 test sessions (criteria used were: 8, 6, 4, 2). Once the criterion was met, the rat received the other stimulus as a sample until it reached the criterion again; stimulus presentations alternated in this way for the duration of the session. The limited hold was reduced to 15 s. For this phase and all remaining phases, a time-out of 20 s was added for an incorrect or no response.

During the final phases of training, stimuli were presented in randomized blocks of six trials (three each of NaCl and DW). The probability of receiving NaCl as a stimulus for these and all following sessions was P = 0.5. For Random Training I, the limited hold was reduced to 10 s. For Random Training II, the limited hold was reduced to 5 s. Rats remained in this phase of training until all rats reached a criterion performance of ≥80% correct overall on trials with a response during at least one session, which occurred after 8 sessions.

Once Testing began, stimulus test sessions were conducted on Tuesday and Thursday of each week, during which the concentration tested was subsequently lowered across sessions by approximately 0.33 log_10_ units from 0.4 M NaCl until all animals had reached chance levels of performance for at least one concentration, which occurred at 0.4 mM NaCl. On Monday, Wednesday and Friday, stimulus control sessions were conducted wherein the stimulus concentration used was the lowest at which an animal had consistently performed ≥80% overall on trials with a response. In this way, stimulus control was tailored to each animal’s sensitivity and performance.

**Water Control Tests**. Following the end of both NaCl threshold testing and discrimination testing (see below), a Water Control Test was conducted to confirm that rats were not using nonchemical cues to guide performance. During this test all sample tubes were filled with DW and half were assigned to either reinforcement ball. The expected outcome of this test is that all animals perform at chance levels (e.g., 50% overall on trials with a response) because there is no stimulus signal to guide the animal which way to respond.

**KCl Detection**. Following the first Water Control Test, rats were trained and tested in a KCl detection task to assess sensitivity to the stimulus, similarly to that conducted for NaCl. Training followed an abbreviated schedule because the animals were already familiar with the operant task. The limited hold was 5 s, and the time-out was 20 s throughout training and testing of KCl sensitivity.

Rats received 2 sessions of Side Training with either 0.4 M KCl or DW as a sample within a single session each, and the rats had access to only the reinforcement ball assigned to that stimulus. The assignments for KCl were the same as assignments for NaCl.

During Random Training, stimuli were presented within a single session in randomized blocks of six trials (three each of KCl and DW). After 4 days, it was determined that some GCX rats were struggling to reach the criterion of ≥80% overall on trials with a response. The training concentration was increased to 0.8 M KCl for all animals. After 2 days of training, most of the rats, regardless of group, were no longer taking all of their sample licks, possibly due to the concentration being so high as to be aversive. Consequently, the training concentration was lowered to 0.6 M KCl, where it remained for 2 days until all rats had reached the criterion level of performance and were taking all 10 sample licks. Because all animals were performing at high levels by the end of training, it is unlikely that adjusting training concentration had adverse effects on the animals’ ability to perform the task.

For KCl testing, as with the NaCl detection test, the KCl concentration was systematically lowered by approximately 0.33 log_10_ units across Tuesday and Thursday sessions until all rats reached chance levels of performance, which occurred at 2.5 mM KCl. The stimulus control sessions were run as described above for NaCl, using the lowest KCl concentration at which the animal consistently performed ≥80% overall on trials with a response.

**Salt Discrimination**. In this phase, rats were trained and tested on their ability to distinguish NaCl from KCl. In this task, the concentration of each stimulus is varied within a single session and the animal is required to distinguish between two similar stimuli independent of stimulus intensity, rather than to simply detect the presence of either chemical, making it a more difficult sensory task compared with the previous phases. Training was an abbreviated form of that described for NaCl detection testing. The limited hold was 5-s and the time-out was 20-s throughout training and testing.

Rats received Side Training, with either 0.4 M NaCl or 0.6 M KCl serving as the stimulus within a session for 2 sessions each with access to only the reinforcement ball assigned to that stimulus. For this test, the reinforcement ball previously assigned to DW was assigned as NaCl, and that assigned as KCl remained so.

For Random Training I, rats received both 0.4 M NaCl and 0.6 M KCl as stimuli within a single session in randomized blocks of 6 trials (three each of NaCl and KCl). This phase continued until all rats had reached a level of performance ≥80% correct overall on trials with a response, which occurred after 10 sessions. For Random Training II, rats were presented with an array of NaCl and KCl concentrations within a single session. These concentrations were presented in randomized blocks of 6 trials (1 trial each of 6 stimulus concentrations). Concentrations were chosen based on the dynamic range of SHAM and GCX mean performance curves calculated for NaCl and KCl (see below) in an attempt to match as best as possible for intensity between stimuli. Specifically, the training concentrations (0.4 M NaCl and 0.6 M KCl) were used for both surgical groups, along with those concentrations at which the average for each surgical group was 80 and 90% overall, calculated independently for each group. Rats within each surgical group were tested with their respective concentrations. Thus, for SHAM rats, the concentrations used were 0.003, 0.006, and 0.4 M NaCl and 0.04, 0.06, and 0.6 M KCl. For GCX rats, the concentrations used were 0.01, 0.02, and 0.4 M NaCl, and 0.09, 0.12, and 0.6 M KCl.

After 4 sessions in Random Training II, it appeared that all rats were struggling to meet the performance criterion, including SHAM rats. Because previous studies have shown that this discrimination is easily learned by rats [39–42], concentrations were raised for all rats to asymptotic levels. The final concentrations tested were 0.1, 0.2, 0.4 M NaCl, and 0.15, 0.3, 0.6 M KCl. Performance immediately improved for all rats and testing continued for six sessions. Because all animals were performing at high levels by the end of training (see below), it is unlikely that adjusting training concentrations had adverse effects on the ability of the animals to perform the task.

**Histology**. After 3–10 days of rehydration following the final Water Control Test, rats were deeply anesthetized with the euthanasia agent Sleepaway (26% sodium pentobarbital, administered at least 60mg/kg, ip; Fort Dodge Animal Health) and transcardially perfused with 0.1 M PBS followed by 10% buffered formalin. Brains were removed and placed in a solution of 10% glycerol in buffered formalin for cryoprotection and stored for slicing. The GC and surrounding areas of insular cortex were sliced into 50 μm coronal sections on a freezing stage microtome, postfixed in formalin for up to 1 h and then rinsed in PBS before being mounted on gelatin-subbed slides and Nissl-stained with thionin.

**Lesion analysis**. The lesions were semi-quantitatively analyzed, by an observer unaware of the specific surgical and injection condition of the rat, with the use of a ternary scoring system on a custom grid which divided our region of interest (GC and the surrounding areas of insular cortex) into subdivisions based on a stereotaxic atlas [43]. This method provided a high-spatial-resolution reconstruction of each lesion, with each row indicating a 50 μm slice and each column devoted to one of the appropriate subdivisions [26].

On the collective basis of reported thalamic projections and the position of taste-responsive neurons [3–5, 44], GC was defined as encompassing the dysgranular (DI) and agranular (AI) subdivisions of the insular cortex dorsal to the rhinal fissure, from +0.2mm to +2.3mm relative to bregma, and bordered medially by the claustrum. We also defined the center region of the taste-responsive cells in DI and AI, falling between +0.6 and +1.8mm from bregma, as the “core”. This region is the cardinal target of recent electrophysiological recording studies of taste-responsive neurons in awake behaving rodents [45–49]. The approximate center of GC, at +1.2mm, separated what we operationally defined as anterior and posterior GC.

To minimize experimental error in the assessment of lesion size and location across animals, associated in part with histological processing, we standardized each brain to the stereotaxic atlas using anatomical landmarks. The A/P axis was standardized based on the most rostral section where the anterior commissure crosses the midline (approximately at bregma) and the most rostral section where the corpus callosum joins the cerebral hemispheres (approximately +2.3 mm anterior to bregma). The D/V axis was standardized based on various anatomical landmarks for each slice depending on its location along the A/P axis.

In addition to GC, the analysis grid included the granular subdivision of insular cortex (GI), the claustrum (C), cortex ventral to the rhinal fissure (V) and cortex dorsal to GI (D), and areas medial to the external capsule (M). The areas GI, DI, AI, V and D were further divided into thirds to provide additional refinement in analysis. Because D, V, and M subdivisions represented the borders of our region of interest and did not have an explicit stereotaxically defined boundary, we simply assigned the rough width of the GC for that section for the M subdivision and its height for the D and V subdivision.

These boundaries defined the grid used to map the lesion in its entirety. The atlas was used as a guide when the scope of the lesion made it difficult to determine boundaries. Each grid cell was evaluated for damage and assigned a lesion score based on a ternary scale. If there was a complete lesion in the grid cell, it was assigned a score of 1.0; if at least half of the grid cell contained a lesion, it was assigned a score of 0.5. If less than half of the grid cell contained a lesion, then it was assigned a score of 0. Both hemispheres were analyzed independently.

We calculated hemispheric symmetry of the lesions for each rat by comparing each corresponding grid cell for the hemispheres and assigning the lesser of the two scores. If both hemispheres had a score of 1.0 for a given grid cell, the symmetry score was 1.0. If the grid cell for one hemisphere was 1.0 and the other 0.5, or if both were 0.5, the symmetry score was 0.5. If the grid cell for one hemisphere had a score of 0.5 or 1.0 and the other 0, or if both were 0, the symmetry score was 0.

The scores from the hemispheric symmetry maps from GCX animals meeting the lesion criteria were used to calculate the average lesion score for each grid cell of the symmetry maps. An aggregate Overlap Map was created from these average values to visually depict the spread and variability in lesions.

**Data analysis**. As the primary goal of this study was to determine the necessity of GC for normal salt detectability and discriminability, only those GCX rats with large bilateral lesions (destroying at least 50% of GC and 70% of the “core” in each hemisphere) were included in behavioral analyses. These criteria were used to be consistent with our prior published studies [26,30] and ensure that the lesions were well placed with a large portion of GC, and in particular the subregion with a high density of taste-responsive neurons, bilaterally damaged. Injection conditions from the CTA phase within a surgical group were combined for analyses of performance on detection and discrimination tasks.

For the brief-access CTA retention test, a Taste/Water Ratio for each stimulus was calculated for each rat:

$$\text{Taste / Water Ratio = }\frac{\text{Mean Licks to Taste Stimulus}}{\text{Mean Licks to Water}}$$

A ratio of 1.0 indicates that the licks to the stimulus matched the licks to water, and therefore no avoidance, whereas ratios approaching 0 signify avoidance of the taste stimulus. Accordingly, the response of each rat to the stimulus was standardized to average licks to water, which under water-deprived conditions tends to elicit near-maximal licking. As such, this ratio adjusts for individual differences in levels of thirst or oromotor competence [36]. Interlick interval, the number of water licks per trial during training, trials initiated on the test day, and each Taste/Water Ratio were compared across surgical and injection groups in ANOVAs.

A CS Preference Ratio for the two-bottle preference test was calculated for each rat, in which CS intake was divided by total fluid intake during the entire 48-h period. A ratio of 0.5 represents equal intake of the CS and DW, while a score of 1.0 indicates that all intake was of the CS (i.e., preference). These ratios were analyzed by appropriate ANOVAs.

For the NaCl and KCl detection data, average proportion correct on trials with a response was calculated for each concentration from all stimulus control and test sessions. For NaCl detectability training, proportion correct was also analyzed for Alternation on trials with a response after a switch between stimuli and for Random Training I on all trials with a response, to determine whether GCX rats were impaired in learning the task. Additionally, a nonlinear logistic equation representing performance as a function of concentration was fit to the overall proportion correct from the detection test sessions:

$$f\left(x\right)=\left[\frac{a-0.5}{\left(1+10^{\left(log_{10}x-c\right)b}\right)}\right]+0.5$$

where x = stimulus concentration in log_10_ units, *a* = asymptotic performance, *b* = slope, and *c* = log_10_ stimulus concentration at ½-asymptote (i.e., EC_50_; operationally defined as the detection threshold).

Overall proportion correct on trials with a response was calculated for each day of Discrimination Training I and analyzed using *t*-tests. Overall proportion correct on trials with a response was collapsed for each of: Discrimination Training I, Discrimination Training II, and Discrimination Testing. These were analyzed with one-way ANOVAs with repeated measures.

For the Water Control Tests, the performance of each rat was tested against chance (i.e., 50% correct overall) in a binomial distribution test.

We compared the degree of GC lesion-induced deficits between tasks by using Pearson’s correlation procedure in which the data for rats with lesions, regardless of whether they met the histological inclusion criteria, were converted to z-scores relative to the mean and standard deviation (SD) of the data for the appropriate SHAM group. The conventional p ≤ 0.05 was used as the criterion for rejecting the null hypothesis in statistical tests.

Three GCX rats died during surgery or shortly thereafter. Additionally, two rats in the GCX-LiCl group were removed from study during NaCl training due to irregular lick topography. Their data are not included in any phase of the experiment.

## Results

**Fig 2:**
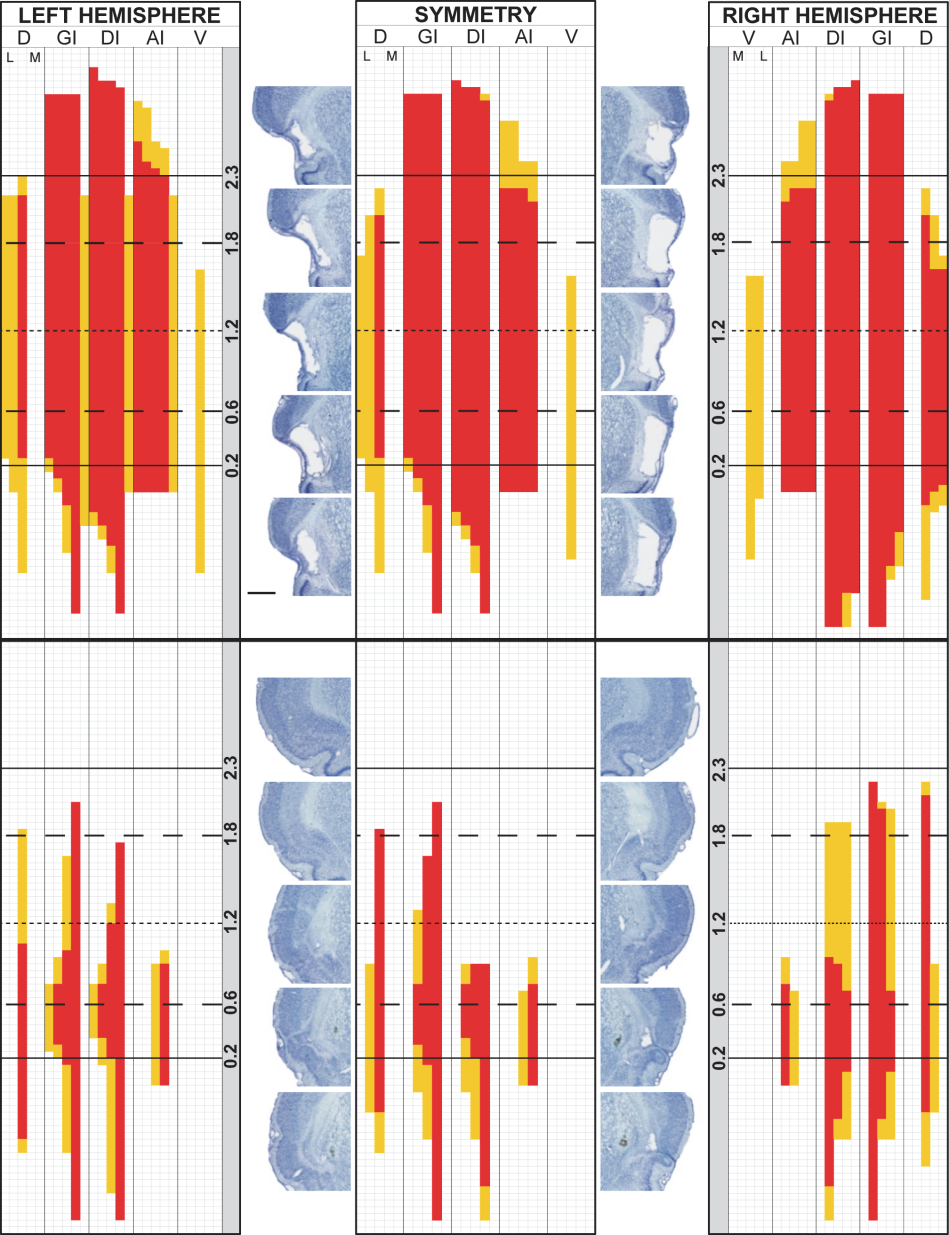
Symmetry Maps for exemplar lesions. Top: a map depicting scoring for the large bilateral lesion of a rat whose lesion met the inclusion criteria for behavioral analyses (≥50% of GC, and ≥70% of the GC “core”, containing a lesion). Bottom: a map depicting scoring for the small bilateral lesion of a rat whose lesion did not meet the inclusion criteria, and was therefore removed from behavioral analyses. Left and right hemispheres are shown, along with the resulting Symmetry Map (middle panel for both exemplars). A score of 1.0, indicating complete destruction of an area (one grid cell), is shown in red. A score of 0.5, indicating at least half of an area destroyed, is shown in orange. A score of 0, indicating less than half destruction of the area, is shown in white. Each row represents a single 50 μm section. Solid horizontal lines (at 2.3, 0.2) indicate anterior and posterior boundaries of the conventionally defined GC. Long-dashed horizontal lines (at 1.8, 0.6) indicate anterior and posterior boundaries of GC “core”. Short-dashed horizontal line (at 1.2) indicates the approximate center of GC. Subdivisions representing the dorsal/ventral axis are divided vertically by solid line; D: cortical areas dorsal to granular insular cortex. GI: Granular insular cortex. DI: Dysgranular insular cortex. AI: Agranular insular cortex, dorsal to the rhinal fissure. V: cortical tissue ventral to the rhinal fissure. Each column of the map within eachdorsal/ventral subdivisions represents a portion of tissue from the lateral (L) to medial (M) direction. Representative photomicrographs for labelled coordinates (in mm, relative to bregma) are shown, in order, for each hemisphere. Scale bar = 1 mm.

**Fig 3:**
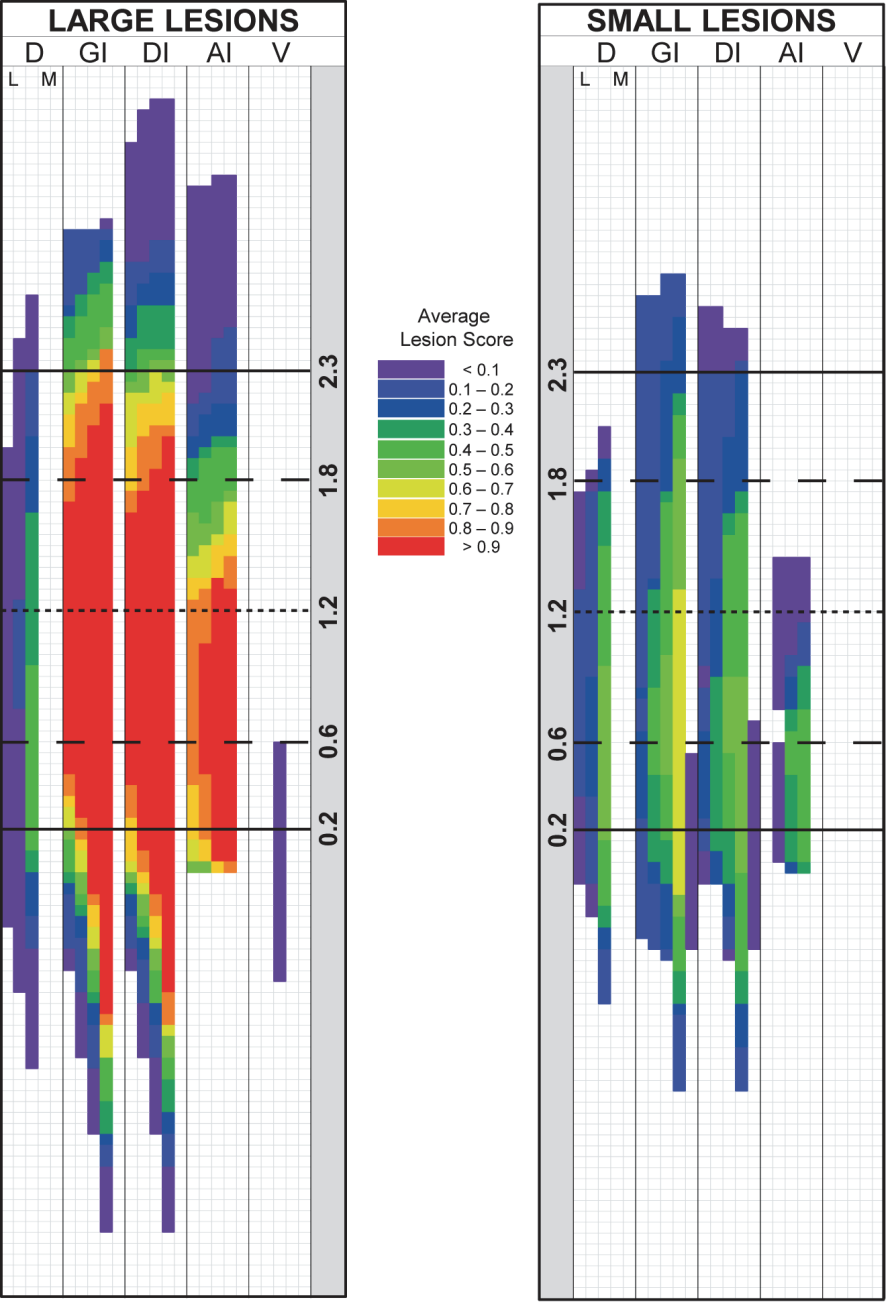
Overlap Map for lesions of rats included in behavioral analyses. A graphic depiction of compiled Symmetry Maps of those rats meeting the inclusion criteria for behavioral analyses (left panel; ≥50% of GC and ≥70% of the GC “core” containing a lesion; n = 17) and lesions too small for inclusion (right panel; n = 9). The lesions for the included rats encompassed 80% of GC and 91% of GC “core” on average. Each color represents the average lesion score for the group in each cell, as shown in the color key to the right of the Overlap Map. Each row represents a single 50 μm section. Solid horizontal lines (at 2.3, 0.2) indicate anterior and posterior boundaries of the conventionally defined GC. Long-dashed horizontal lines (at 1.8, 0.6) indicate anterior and posterior boundaries of GC “core”. Short-dashed horizontal line (at 1.2) indicates the approximate center of GC. Subdivisions representing the dorsal/ventral axis are divided vertically by solid line; D: cortical areas dorsal to granular insular cortex. GI: Granular insular cortex. DI: Dysgranular insular cortex. AI: Agranular insular cortex, dorsal to the rhinal fissure. V: cortical tissue ventral to the rhinal fissure. Each column of the map within eachdorsal/ventral subdivisions represents a portion of tissue from the lateral (L) to medial (M) direction.Scale bar = 1 mm.

After lesion analysis, 17 of the 26 GCX rats that completed the study met the lesion criteria (≥50% of GC, and ≥70% of GC “core”, containing a lesion) and therefore were included in behavioral analyses. Fig. 2 displays two representative bilateral lesions: a large lesion that met the lesion criteria (top) to be included in behavioral analyses and a small lesion (bottom) that did not. Each hemisphere was scored as described above (left- and right-most grids) resulting in individual Symmetry Maps (center grids). Photomicrographs of each hemisphere at specific A/P coordinates relative to bregma are provided for comparison with the analysis grid. An Overlap Map (Fig. 3, left) depicting the lesions for all rats included in the behavioral analyses shows that the lesions were large, encompassing 80% of GC and 91% of the GC “core” on average. The lesions extended beyond the GC and encroached upon the claustrum, the overlying granular layer of insular cortex, and some portion of the insular cortex both anterior and posterior to the GC. An Overlap Map of lesions too small to be included in behavioral analyses is also provided (Fig. 3, right). These animals had lesions that symmetrically encompassed, on average, only 18% of the GC and 26% of the GC core. Final group sizes for behavioral analyses were: SHAM-NaCl, n = 7; SHAM-LiCl, n = 7; GCX-NaCl, n = 8; GCX-LiCl, n = 9. Injection conditions were combined within surgical groups for detection threshold and discrimination analyses.

### CTA Retention Tests

**Fig 4:**
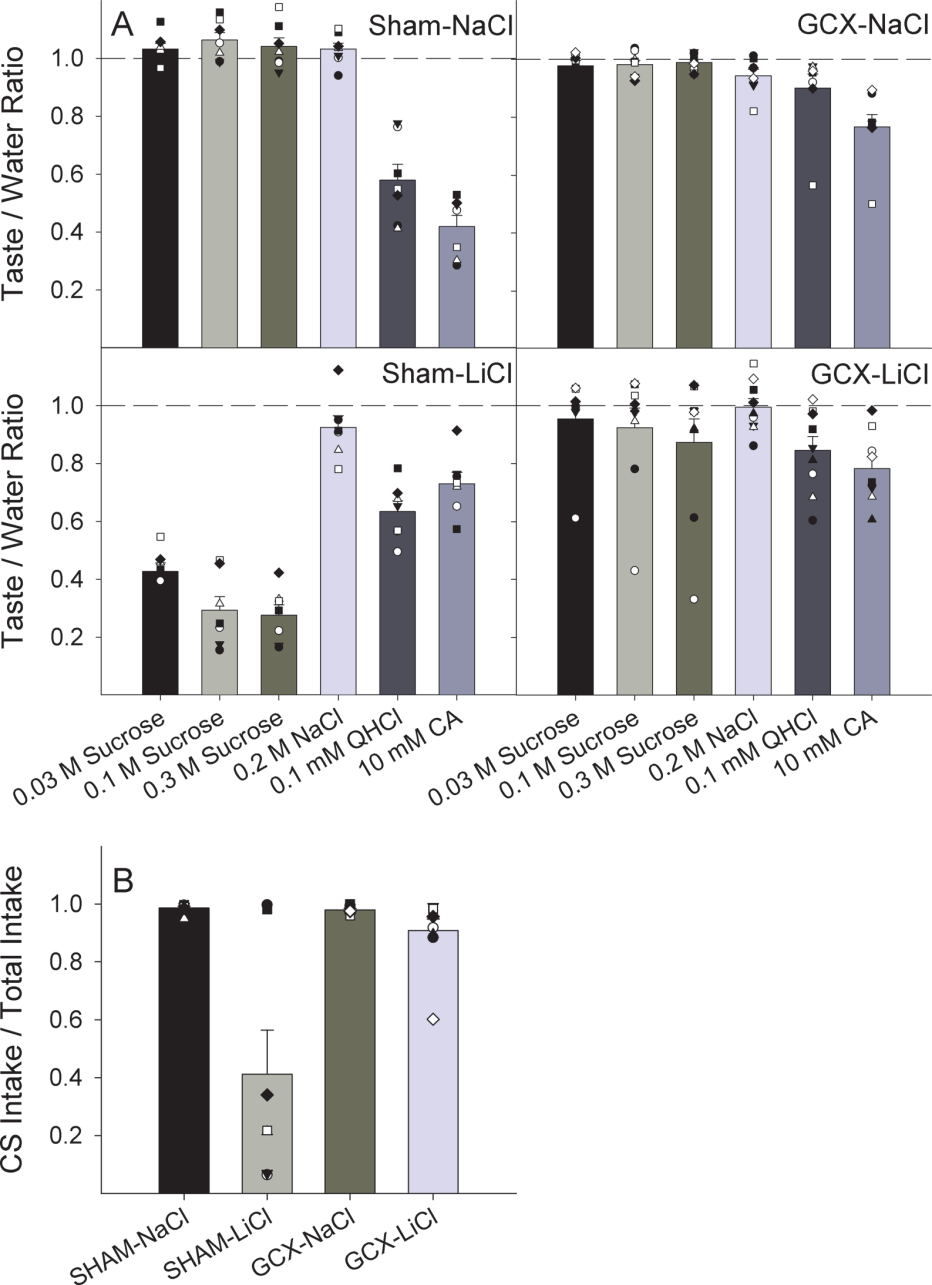
Postsurgical assessments of presurgically conditioned taste aversion to sucrose. Panel A: Mean (±SE) Taste/Water Ratios for each stimulus by group. A score of 1.0 (dashed line) indicates licking rates comparable to water; a score approaching 0 indicates avoidance of the stimulus. Plotted points are individual animals’ scores within each group. Panel B: Mean (±SE) group preference scores during the 48-h 2-bottle intake test, comparing CS intake to total intake. A score of 1.0 indicates total preference for the CS; a score approaching 0 indicates total avoidance of the CS. Plotted points are scores for individual animals within the group.

On average, the GCX-LiCl group did not postsurgically express the presurgically conditioned aversion to sucrose during the brief-access retention test (Fig. 4A). Two-way ANOVAs conducted for each stimulus revealed main effects from surgery and injection as well as a surgery x injection interaction for all concentrations of sucrose (Table 2), seemingly driven by lower Taste/Water Ratios from the SHAM-LiCl group relative to the GCX-LiCl group (see Fig. 4A). Additionally, there were significant effects for the NaCl, quinine, and citric acid stimuli; these differences appear to be generally driven by the higher Taste/Water Ratios in both groups of GCX rats, at least for quinine and citric acid. These results cannot be explained simply by a stronger drive to drink by GCX rats while water-deprived (i.e., hyperdipsia) because no differences were seen in trials taken on the test day, or licks taken to water during training, or total fluid consumption during the two-bottle preference tests (Tables 2 & 3). Also, use of the Taste/Water ratio adjusts for individual differences in levels of thirst or oromotor competence, so these results are not explained by the slight but significant reduction in the local licking rate in the GCX groups (Table 2). It is possible, however, that these stimuli were weaker to GCX rats as suggested by the explicit tests of the perithreshold sensitivity to NaCl and KCl (see below). If a Bonferroni correction is made adjusting for the separate tests conducted for each stimulus, all effects would remain significant except for the surgery x injection interaction for NaCl.

**Table 2:** Two-way ANOVAs for CTA retention testing.

Taste/Water Ratios during CTA Retention Test			
	Surgery	Injection	Surgery x Injection
Sucrose			
0.03 M	F(1,27) = 51.0, **p<0.001**	F(1,27) = 89.1, **p<0.001**	F(1,27) = 76.3, **p<0.001**
0.1 M (CS)	F(1,27) = 33.2, **p<0.001**	F(1,27) = 75.2, **p<0.001**	F(1,27) = 55.6, **p<0.001**
0.3 M	F(1,27) = 26.9, **p<0.001**	F(1,27) = 70.1, **p<0.001**	F(1,27) = 38.2, **p<0.001**
0.2 M NaCl	F(1,27) = 0.1, p = 0.770	F(1,27) = 0.9, p = 0.355	F(1,27) = 7.2, **p = 0.012**
0.1 mM quinine	F(1,27) = 30.5, **p<0.001**	F(1,27)<0.1, p = 0.999	F(1.27) = 1.3, p = 0.261
10 mM citric acid	F(1,27) = 23.8, **p<0.001**	F(1,27) = 15.9, **p<0.001**	F(1,27) = 12.9, **p = 0.001**
Session parameters			
	Surgery	Injection	Surgery x Injection
Test trials taken	F(1,27) = 0.2, p = 0.640	F(1,27) = 2.7, p = 0.110	F(1,27)<0.1, p = 0.859
Total licks^1^	F(1,27) = 0.1, p = 0.723	F(1,27)<0.1, p = 0.968	F(1,27) = 0.4, p = 0.548
ILI^1^ ^,^ ^2^	F(1,27) = 13.8, **p<0.001**	F(1,27) = 0.7, p = 0.410	F(1,27) = 1.7, p = 0.128
Two-bottle intake test			
	Surgery	Injection	Surgery x Injection
48-hr preference	F(1,27) = 11.2, **p = 0.002**	F(1,27) = 19.4, **p<0.001**	F(1,27) = 11.8, **p = 0.002**
CS intake (ml)	F(1,27) = 0.4, p = 0.547	F(1,27) = 16.1, **p<0.001**	F(1,27) = 2.4, p = 0.129
Total intake (ml)	F(1,27)<0.1, p = 0.897	F(1,27) = 12.2, **p<0.002**	F(1,27) = 0.9, p = 0.36

Significant p-values indicated in bold.

^1^: From the final day of stationary training

^2^: Interlick interval (in ms)

**Table 3:** Brief-access training and testing means (SE) by group.

	SHAM-NaCl	SHAM-LiCl	GCX-NaCl	GCX-LiCl
Stationary				
Licks	2794 (251)	2962 (220)	2868 (350)	2676 (302)
ILI^1^	156.0 (1.7)	154.7 (2.2)	162.4 (2.1)	168.1 (3.5)
Water Training				
Trials	91.0 (7.1)	91.9 (5.7)	98.8 (7.5)	84.9 (5.3)
Licks	66 (0.9)	65 (1.8)	65 (1.0)	62 (1.6)
Test				
Trials	124.6 (4.8)	116.4 (4.6)	125.9 (4.3)	119.3 (4.1)
Licks to water	63 (1.2)	63 (2.5)	65 (1.3)	59 (1.7)

^1^: Interlick interval (in ms)

Similar to the results from the brief-access CTA retention test, a two-way ANOVA conducted on the 48-hr preference ratio to 0.1 M sucrose showed an effect of surgery, with SHAM-LiCl rats on average drinking less of the CS (Table 2; Fig. 4B); this effect was significant despite the apparent extinction by 2 SHAM-LiCl rats after exposure to the CS during the brief-access test without subsequent negative consequence.

### NaCl and KCl Taste Sensitivity

**Fig 5:**
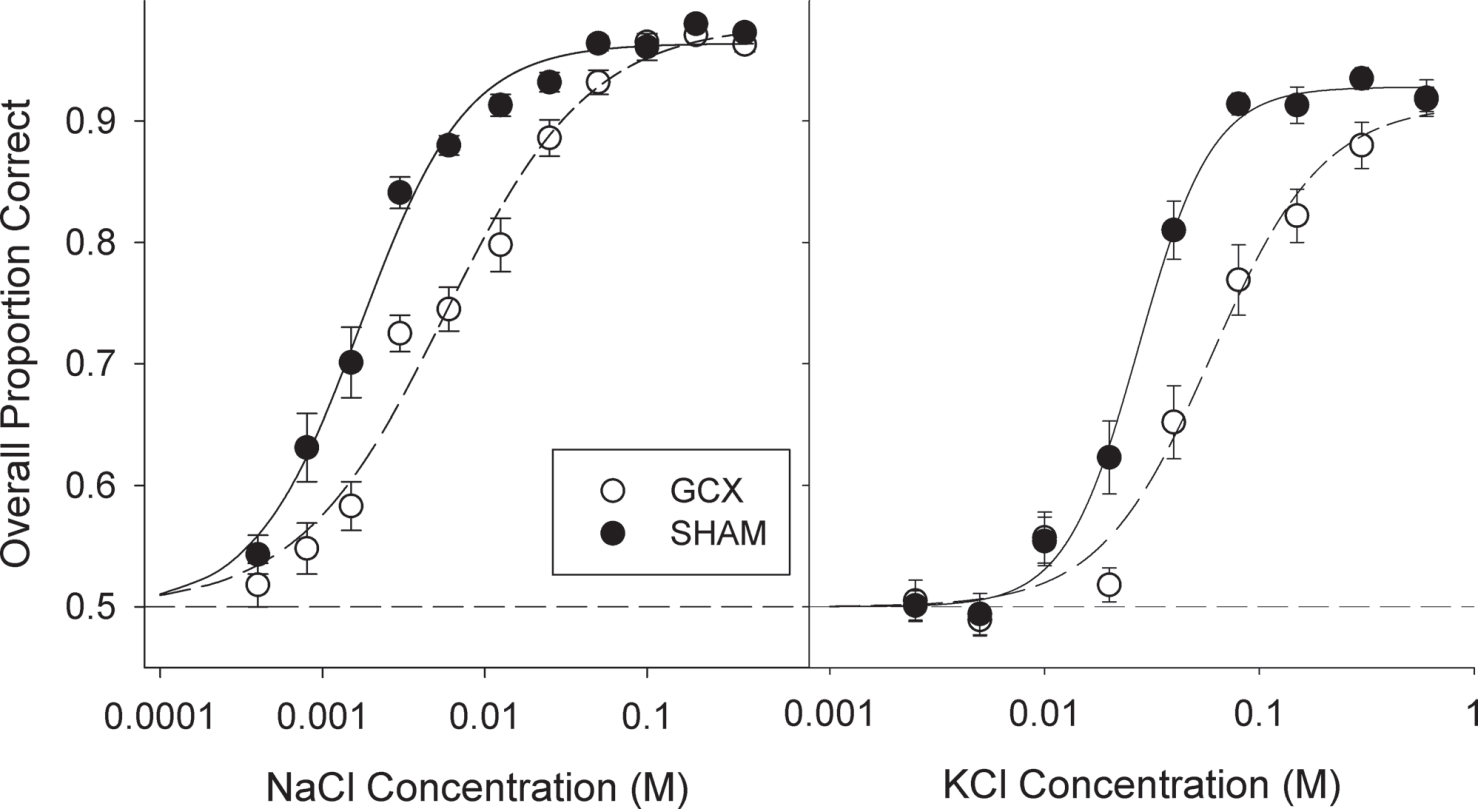
Salt taste detection testing. Mean logistic curves for NaCl (left) and KCl (right) detection threshold testing, derived from overall proportion correct to each stimulus concentration (SHAM: closed circles; GCX: open circles, plotted as group mean ± SE).

For both salts tested, GCX rats that had lesions meeting the criteria for inclusion were, on average, impaired in the detection tasks. Damage to the GC shifted the psychometric sensitivity functions to the right (e.g., decreased sensitivity) relative to SHAM rats (Fig. 5). Two-way ANOVAs showed significant effects for both NaCl and KCl (Table 4), and *t*-tests on the logistic curve parameters showed that GCX rats had a significantly higher EC_50_ (*c*-value) for both salts (Table 5). The GCX rats also displayed significantly higher asymptotic performance (*a*-value) and a shallower slope (*b*-value) for the psychometric NaCl function compared with the SHAM group, but the differences were minor in magnitude and not observed when KCl was the test stimulus (Table 5). Thus, despite displaying lower sensitivity to these salts, GCX rats were fully capable of learning and performing the task when given sufficiently high concentrations. This is further emphasized by the fact that, even during Alternation Training and Random Training I-II for NaCl, GCX rats performed at levels equal to SHAM rats (data not shown; all p-values>0.342).

**Table 4:** Two-way ANOVAs for NaCl and KCl detection testing.

	Surgery	Concentration	Stimulus x Concentration
NaCl	F(1,29) = 29.0, **p<0.001**	F(10,270) = 267.1,**p<0.001**	F(10,270) = 8.0, **p<0.001**
KCl	F(1,29) = 21.1, **p<0.001**	F(8,232) = 191.7, **p<0.001**	F(8,232) = 6.4, **p<0.001**

Significant p-values indicated in bold.

**Table 5:** Mean (SE) of group curve parameters for salt detection testing.

	GCX	SHAM	t-test
NaCl			
a	0.982 (0.004)	0.958 (0.005)	t(29) = 3.494, **p = 0.002**
b	-1.096 (0.089)	-1.941 (0.340)	t(29) = 2.621, **p = 0.014**
c^1^	-2.254 (0.068)	-2.789 (0.054)	t(29) = 5.956, **p<0.001**
KCl			
a	0.928 (0.016)	0.931 (0.006)	t(29) = 0.18, p = 0.859
b	-7.140 (2.596)	-12.378 (3.978)	t(29) = 1.654, p = 0.109
c^1^	-1.174 (0.071)	-1.528 (0.038)	t(29) = 4.149, **p<0.001**

Significant p-values indicated in bold.

^1^Values in log_10_ M concentrations

### Salt Discrimination

**Fig 6:**
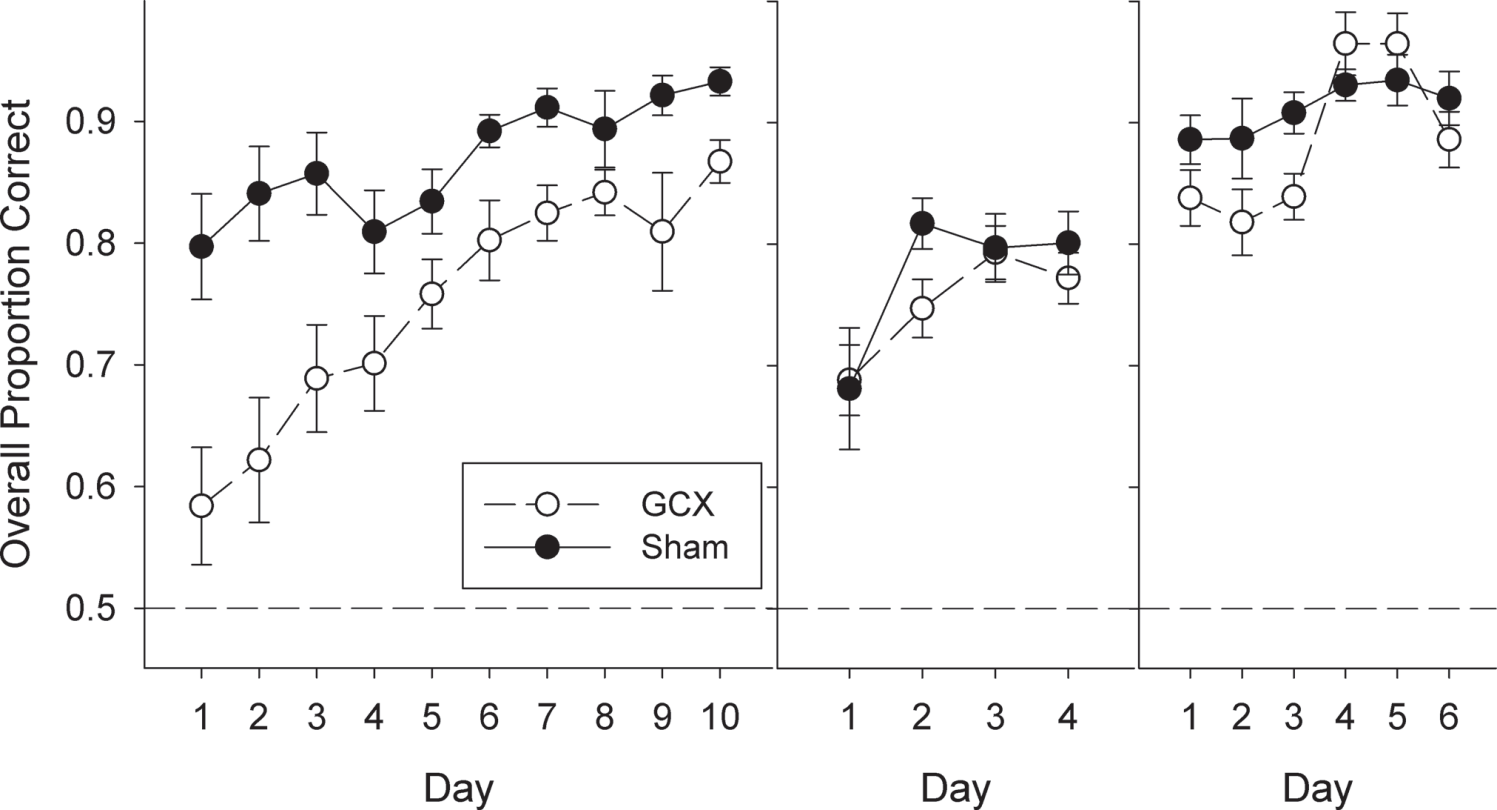
Salt taste discrimination training and testing. Mean (± SE) proportion correct for Discrimination Training I (left), Discrimination Training II (middle), and Discrimination Test (right) for SHAM (closed circles) and GCX (open circles) groups.

Early in training for the discrimination test, GCX rats displayed difficulty in learning the task (Fig. 6, left). During Discrimination Training I, when required to discriminate only one concentration of NaCl from one concentration of KCl, GCX rats were significantly impaired relative to SHAM rats (Table 6). This was evident on the very first session (t = 10.4, p>0.001). However, given time, rats with extensive damage to the GC were able to show competency and met the criterion performance of ≥80% overall on trials with a response. When training became more difficult with the inclusion of multiple concentrations during Random Training II, there was an effect of Day (Fig. 6, middle) but no statistical effect involving surgical condition (Table 6), indicating that both GCX and SHAM animals were similarly affected. However, because the SHAM group was clearly struggling with the chosen concentration set and barely reaching criterion levels of performance on a task easily learned by rats [39–42], the concentrations were increased for all animals. Consequently, performance improved for both surgical groups, with no statistical difference between them (Fig. 6, right; Table 6).

**Table 6:** Two-way ANOVAs for NaCl v KCl discrimination training and testing.

Phase	Surgery	Day	Surgery x Day
Training I	F(1,29) = 9.4, **p = 0.006**	F(9,189) = 9.9, **p<0.001**	F(9,189) = 2.5, **p = 0.011**
Training II	F(1,29) = 0.6, p = 0.422	F(3,87) = 11.3, **p<0.001**	F(3,87) = 1.2, p = 0.298
Testing	F(1,29) = 4.3, p = 0.054	F(5,145) = 4.0, **p = 0.002**	F(5,145) = 0.5, p = 0.801

Significant p-values indicated in bold.

### Water Control Tests

In the Water Control Test conducted after the NaCl detection test, the average performances (±SE) for GCX and SHAM rats were 48.7 (±2.1) and 50.4 (±1.0), respectively; chance is 50% correct. Although one SHAM rat performed statistically above chance on the Water Control Test based on a binomial distribution test, its performance (64%) was relatively poor, and this same animal had reached chance levels to the low concentrations during NaCl testing. For the Water Control Test conducted after discrimination testing, GCX and SHAM rats performed at 52.8 (±1.0) and 50.6 (±1.3), respectively, with no rats individually performing above chance levels. Thus, it is clear that the rats were responding based on the chemical nature of the stimulus and not extraneous cues.

### Behavioral Correlations

**Fig 7:**
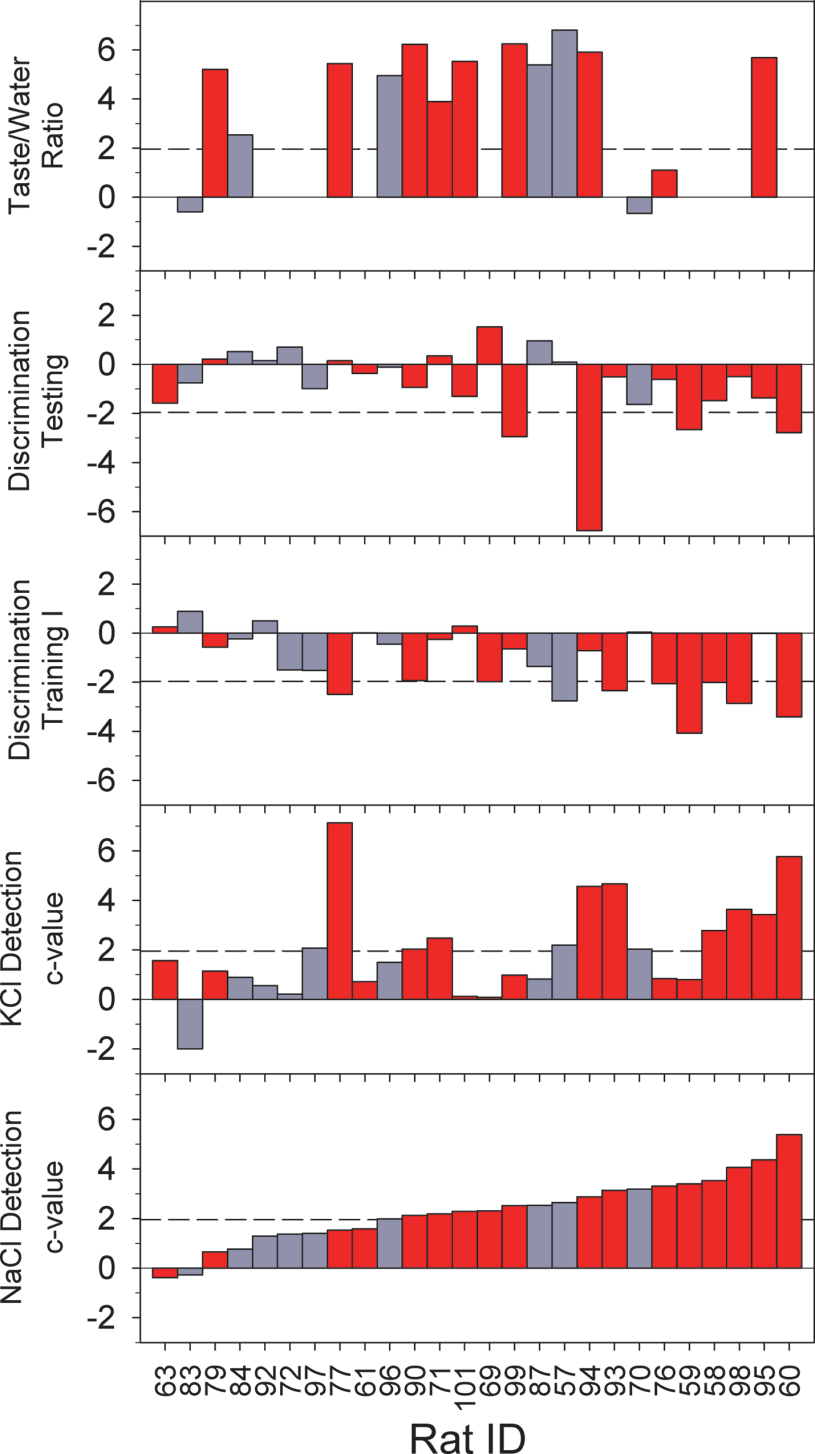
The z-scores for individual GCX rats relative to the SHAM distribution for different behavioral tests. The z-scores calculated are relative to SHAM-LiCl mean and SD for Taste/Water Ratios to 0.1 M sucrose (CS; top), and relative to the full SHAM group for detection testing and discrimination training. Dashed line indicates a z-score of 1.96 (or -1.96 for discrimination phases), the criterion for being classified as impaired on a task. Red bars indicate rats with lesions meeting the inclusion criteria for behavioral analyses (≥50% of GC and ≥70% of the GC “core” containing a lesion).

Although for every task there were some animals, regardless of lesion size and topography, that displayed significant lesion-induced impairments (z-score ≥ 1.96 relative to SHAM group), for the most part impairment on one task did not predict impairment on another (Table 7; Fig. 7). There was a modest correlation between the z-scores of the EC_50_ values for NaCl and KCl in the animals with lesions and significant correlations between performance early (Days 1–3) during Discrimination Training I and EC_50_ values for both detection tasks. This latter result suggests that for some animals the delayed acquisition of the salt discrimination task may have been related to a lesion-induced decrease in the intensity of the taste cues. In contrast, degree of avoidance during the brief-access CTA retention test did not predict impairment for either sensitivity to or discriminability between the salts tested here.

**Table 7:** Pearson correlations between behavioral tests.

	Taste/Water Ratio (to CS)	NaCl (c)	KCl (c)	Discrim Training 1–3
Taste/Water Ratio (to CS)^1^				
NaCl (c)^2^	0.249			
KCl (c)^2^	0.425	0.509		
Discrim Training I 1–3^2^	-0.342	-0.572	-0.461	
Discrim Testing^2^	-0.087	-0.330	-0.317	-0.045

Significant values indicated in bold.

^1^: These correlations were conducted with LiCl-injected GCX rats.

^2^: These correlations were conducted with all GCX rats.

While this study was designed to ascertain the effects of large lesions to GC without systematic variation of lesion placement, there was some variation in lesion size and placement between animals. Further comparisons of the lesion variability between subgroups of GCX rats (those impaired vs. those GCX rats unimpaired relative to the SHAM distributions for each behavioral task) did suggest some modest spatial segregation with respect to taste function but its interpretive value is limited because the experiment was not designed to generate systematic variability in lesion location and size. As such, those comparisons are not presented here.

## Discussion

It is evident from the results presented here that the region of insular cortex encompassing the GC is necessary for the maintenance of normal taste sensitivity to NaCl and KCl as assessed in animals with bilateral excitotoxic lesions to the area. Likewise, lesions in this brain region retard the acquisition of a NaCl vs. KCl taste discrimination and compromise the postsurgical expression of a presurgical CTA to sucrose. At the same time, however, the animals with histologically confirmed lesions of the GC, while displaying these clear impairments, also displayed an impressive degree of competence in the psychophysical tasks. The functional implications of these outcomes are discussed in detail below.

### Insular Cortex Lesions Impair Retention of a Presurgical CTA

In agreement with prior studies in the literature, large bilateral lesions within insular cortex that were focused on GC disrupted the retention of a presurgical CTA [2; 8–12]. This contrasts with two recent studies from our laboratory in which rats with extensive lesions of the conventionally-defined GC displayed normal postsurgical retention of a presurgical CTA to 0.1 M NaCl [26,30]. However, in those studies, two conditioning trials occurred presurgically, whereas here there was only one. Thus, the potentially weaker aversion in the LiCl-injected rats of this study may have been more vulnerable to GC damage. Schier et al. (2014) systematically varied lesion size and placement and found that while, as a group, rats with extensive damage of GC displayed normal retention of a presurgical CTA, some of those animals showed significant impairments. Likewise, some animals not meeting the lesion inclusion criteria (as used here) were impaired in the task. When Schier et al. (2014) employed the same lesion mapping strategy as used here to compare impaired GCX rats to unimpaired GCX rats, the impaired animals had lesions that encompassed posterior GC and extended dorsal and caudal to this area. Interestingly, most of the animals in the GCX-LiCl group in the present study had damage that included this same region of insular cortex. Thus, the disparity in results between this study and the previous work from our laboratory may be more apparent than real and related to the exact topography of the cortical damage.

Indeed, the difference in the relative extent of involvement of this critical cortical region across studies possibly explains the inconsistency in the literature regarding the effectiveness of manipulations of GC activity, either through lesions or pharmacological means, to disrupt CTA expression [10–11,13,18,21–25,29,50–51]. It would be instructive to target the hotspot identified by Schier et al. (2014) with more selective lesions to determine whether disruption of this area alone is sufficient to lead to CTA impairments or whether other cortical areas must be damaged as well.

It is important to acknowledge that the functional basis for the deficit in CTA retention as a result of the lesions here remains unclear. It could represent a problem with taste memory, US memory, or conditioned response expression. It might also result from decreased intensity or recognition of the conditioned stimulus, which is unknown without explicitly testing sucrose detection sensitivity [52]; certainly, such an explanation would not be surprising given the effect on NaCl and KCl sensitivities (Fig. 5).

### Insular Cortex Lesions Decrease Salt Taste Sensitivity and Discriminability

Additional testing revealed that extensive damage of the GC impaired both detection of and discrimination between two common salt stimuli. This is consistent with reports that humans display hypogeusia and have difficulty recognizing taste stimuli after damage to the insular cortex [32–35,53–54], and implicates GC in maintaining normal salt taste sensitivity. At the same time, it is clear that the GC is not necessary for rats to detect midrange and higher concentrations of NaCl and KCl. It was only as the detection task became more difficult with decreasing concentrations that the GCX rats began to display behavioral deficits. Importantly, rats were both trained and tested postsurgically, and there were no differences between the performance of GCX and SHAM rats during NaCl detection training using a high stimulus concentration. Thus, while GC is necessary for normal salt sensitivity to be maintained, it is not required to allow an animal to learn, remember, or express the cue-response-consequence association attendant with the operant taste detection task used here.

An effect of GC lesions on taste learning was revealed, however, when animals were asked to discriminate between salts rather than merely detect their presence. Initially, even with only one high concentration of each salt, GCX rats performed significantly poorer than SHAM rats and showed retarded acquisition of the discrimination. Given time, however, GCX rats were able to reach performance levels comparable to SHAM rats (Fig. 6). Because GCX animals readily learned the rules of the two-response task, it is unlikely that their initial lack of competence in the discrimination task represented a general deficit in learning. Instead, it is likely due to a disruption in the ability to recognize and differentiate between the tastants, even though the animals were able to eventually meet the sensory challenge. It is possible that even the clearly detectable NaCl and KCl concentrations (Fig. 5) used to initially train the discrimination may have still been less intense to GCX rats compared with the SHAM animals, and this may have contributed to the slower acquisition in the former group. The signal detection task used here does not provide explicit information regarding perceived intensity at suprathreshold concentration values.

The underlying mechanism that allows animals to eventually learn the discrimination and perform it normally is unclear. The current study shows, however, that the conventionally defined GC is not necessary for competence on this task to be displayed. Thus, the remaining resources of the gustatory system are capable of supporting this discriminative taste function. The fact that sensitivity to the salt compounds tested here is compromised strongly suggests that the GC contributes to the detection of taste stimuli, but other structures in the central gustatory system must also be involved. It is well established that the GC forms reciprocal connections with other taste-related forebrain sites such as the amygdala, the thalamus, and the lateral hypothalamus and also sends descending projections to the gustatory zone of the parabrachial nucleus in the brainstem [55–59]. Lesions in the PBN severely disrupt sensitivity to taste compounds including NaCl [52,60–61]. Lesion studies are designed to assess the necessity of the neural sites targeted with respect to a specific tested function. Thus, it is quite possible that damage to other forebrain sites receiving taste input could lead to similar partial impairments in sensitivity and discrimination learning as described here, even when thalamic input to GC is left intact. It would also follow that combined lesions of GC with other forebrain taste sites such as the amygdala might lead to more severe effects on taste sensitivity in rats that emulates what is seen after PBN lesion. These possibilities remain to be tested, with the hypothesis being that simple detection of and discrimination between taste compounds involves a distributed process across a variety of forebrain sites. Indeed, interactions between the amygdala and GC in taste processes have been noted electrophysiologically [62–64].

### Functional Role of GC in Taste-Guided Behavior

While the performance of those rats with large lesions was collectively impacted across all taste-guided behaviors tested here, there was only a moderate correlation between the degrees of lesion-induced impairment displayed for each measure. Also, some animals with small lesions whose data were not included in behavioral analyses showed impairment in one or more tasks. This is reflected in the fact that the deficits in behavioral performance, as measured by z-scores relative to the SHAM distribution, did not significantly correlate between all tasks (Table 7; Fig. 7). If the entire region of insular cortex designated as GC were to contribute equally to normal maintenance of all behaviors tested, it might be expected that similar levels of impairment would be seen across behaviors for a given animal (and thus result in significant correlations). However, while performance was affected collectively across all tests for animals with large lesions, these correlations show that on an individual basis the level of impairment could vary from one task to the next. The implication of these results is that subregions within and around GC may contribute differentially to these behaviors, an intriguing possibility also suggested by imaging studies of taste stimulus-evoked activity in rat [6] and mouse insular cortex [7], and that idiosyncratic individual differences in the lesions and/or the topographic representation of function contributed to the relative lack of covariance. In this sense, the use of a lesion mapping strategy as employed here coupled with experiments designed to systematically promote variation in location and size (unlike in this study) could aid in identifying specific cortical regions associated with particular gustatory functions that could then be selectively targeted with lesion or optogenetic manipulations. It is important to note that the results reported here do not contradict the conventional definition of GC, largely determined by electrophysiological data and considered the region of insular cortex that responds after oral taste bud fields are stimulated by chemicals. Rather, our findings contribute to a more functionally oriented mapping of this region of insular cortex which can be compared with what is known based on electrophysiological and imaging studies of taste-related neural activity.

Is the functional competence or incompetence of rats with massive lesions to the gustatory cortex more impressive? This study represents the first explicit test, to our knowledge, of the role of GC in taste detection and discrimination without use of a method that depends on the hedonic properties of the stimulus to drive responding. On the one hand, it provides some support for an early hypothesis postulated by Pfaffmann and colleagues (1977) suggesting that the GC subserves taste stimulus identification and recognition, while the ventral forebrain limb of the central gustatory pathway diverging from the PBN in rodents is more involved with processes associated with taste-related motivation [31]. On the other hand, massive bilateral damage to the GC does not by any means completely eliminate the ability of rats to detect and discriminate the taste stimuli tested here. The tasks employed in our study are not trivial but they may, nonetheless, be sufficiently simple for the remaining resources of the gustatory system to maintain a reasonable degree of competence. In the detection tasks, the animals were only asked to discriminate a taste from water and in the salt discrimination task the animals needed only to discriminate between two taste stimuli. We speculate that GCX animals would have a much harder time detecting target compounds in more complex backgrounds as well as discriminating between chemical mixtures as is generally required in the wild, a possibility that awaits further experimental scrutiny.

## References

[1] Benjamin RM, Pfaffmann C (1955) Cortical localization of taste in albino rat. J Neurophysiol 18:56–64. 13222157 10.1152/jn.1955.18.1.56

[2] Yamamoto T, Matsuo R, Kawamura Y (1980) Localization of cortical gustatory area in rats and its role in taste discrimination. J Neurophysiol 44:440–455. 7441309 10.1152/jn.1980.44.3.440

[3] Kosar E, Grill HJ, Norgren R (1986a) Gustatory cortex in the rat. I. Physiological properties and cytoarchitecture. Brain Res 379:329–341. 3742225 10.1016/0006-8993(86)90787-0

[4] Cechetto DF, Saper CB (1987) Evidence for a viscerotopic sensory representation in the cortex and thalamus in the rat. J Comp Neurol 262:27–45. 2442207 10.1002/cne.902620104

[5] Hanamori T, Kunitake T, Kato K, Kannan H (1998) Responses of neurons in the insular cortex to gustatory, visceral, and nociceptive stimuli in rats. J Neurophysiol 79:2535–2545. 9582226 10.1152/jn.1998.79.5.2535

[6] Accolla R, Bathellier B, Petersen CC, Carleton A (2007) Differential spatial representation of taste modalities in the rat gustatory cortex. J Neurosci 27: 1396–1404. 17287514 10.1523/JNEUROSCI.5188-06.2007 PMC6673570

[7] Chen X, Gabitto M, Peng Y, Ryba NJ, Zuker CS (2011) A gustotopic map of taste qualities in the mammalian brain. Science 333:1262–1266. 10.1126/science.1204076 21885776 PMC3523322

[8] Braun JJ, Kiefer SW, Ouellet JV (1981) Psychic ageusia in rats lacking gustatory neocortex. Experimental Neurology 72:711–716. 7238720 10.1016/0014-4886(81)90020-0

[9] Braun JJ, Lasiter PS, Kiefer SW (1982) The gustatory neocortex of the rat. Physiol Psychol 10:13–45.

[10] Kiefer SW, Leach LR, Braun JJ (1984) Taste agnosia following gustatory neocortex ablation: dissociation from odor and generality across taste qualities. Behav Neurosci 98:590–608. 6540588 10.1037//0735-7044.98.4.590

[11] Cubero I, Thiele TE, Bernstein IL (1999) Insular cortex lesions and taste aversion learning: effects of conditioning method and timing of lesion. Brain Res 839:323–330. 10519056 10.1016/s0006-8993(99)01745-x

[12] Stehber J, Simon F (2011) Involvement of the insular cortex in retention of conditioned taste aversion is not time dependent. Neurobiol Learn Mem 95: 14–18. 10.1016/j.nlm.2010.10.002 20955809

[13] Braun JJ, Slick TB, Lorden JF (1972) Involvement of gustatory neocortex in the learning of taste aversions. Physiol Behav 9:637–641. 4599681 10.1016/0031-9384(72)90023-6

[14] Lorden JF (1976) Effects of lesions of the gustatory neocortex on taste aversion learning. J Comp Physiol Psychol 5:665–679. 10.1037/h0077237 950392

[15] Bermudez-Rattoni F, McGaugh JL (1991) Insular cortex and amygdala lesions differentially affect acquisition on inhibitory avoidance and conditioned taste aversion. Brain Res 549:165–170. 1654172 10.1016/0006-8993(91)90616-4

[16] Kiefer SW, Orr MR (1992) Taste avoidance, but not aversion, learning in rats lacking gustatory cortex. Behav Neurosci 106:140–146. 1313241 10.1037//0735-7044.106.1.140

[17] Nerad L, Ramirez-Amaya V, Ormsby CE, Bermudez-Rattoni F (1996) Differential effects of anterior and posterior insular cortex lesions on the acquisition of conditioned taste aversion and spatial learning. Neurobiol Learn Mem 66:44–50. 8661250 10.1006/nlme.1996.0042

[18] Fresquet N, Angst MJ, Sandner G (2004) Insular cortex lesions alter conditioned taste avoidance in rats differentially when using two methods of sucrose delivery. Behav Brain Res 153:357–365. 15265630 10.1016/j.bbr.2003.12.011

[19] Roman C, Nebieridze N, Sastre A, Reilly S (2006) Effects of lesions of the bed nucleus of the stria terminalis, lateral hypothalamus, or insular cortex on conditioned taste aversion and conditioned odor aversion. Behav Neurosci 120:1257–1267. 17201470 10.1037/0735-7044.120.6.1257

[20] Roman C, Reilly S (2007) Effects of insular cortex lesions on conditioned taste aversion and latent inhibition in the rat. Eur J Neurosci 26:2627–2632. 17970726 10.1111/j.1460-9568.2007.05872.x

[21] Geddes RI, Han L, Baldwin AE, Norgren R, Grigson PS (2008) Gustatory insular cortex lesions disrupt drug-induced, but not lithium chloride-induced, suppression of conditioned stimulus intake. Behav Neurosci 122:1038–1050. 10.1037/a0012748 18823161 PMC3684281

[22] Naor C, Dudai Y (1996) Transient impairment of cholinergic function in the rat insular cortex disrupts the encoding of taste in conditioned taste aversion. Behav Brain Res 79:61–67. 8883817 10.1016/0166-4328(95)00262-6

[23] Rosenblum K, Meiri N, Dudai Y (1993) Taste memory: the role of protein synthesis in gustatory cortex. Behav Neural Biol 59:49–56. 8442732 10.1016/0163-1047(93)91145-d

[24] Rosenblum K, Berman DE, Hazvi S, Lamprecht R, Dudai Y (1997) NMDA receptor and the tyrosine phosphorylation of its 2B subunit in taste learning in the rat insular cortex. J Neurosci 17:5129–5135. 9185550 10.1523/JNEUROSCI.17-13-05129.1997 PMC6573317

[25] Bermudez-Rattoni F (2004) Molecular mechanisms of taste-recognition memory. Nat Rev Neurosci 5:209–217. 14976520 10.1038/nrn1344

[26] Schier LA, Hashimoto K, Bales MB, Blonde GD, Spector AC (2014) High-resolution lesion-mapping strategy links a hot spot in rat insular cortex with impaired expression of taste aversion learning. Proc Natl Acad Sci USA 111:1162–1167. 10.1073/pnas.1315624111 24395785 PMC3903191

[27] Benjamin RM (1955) Cortical taste mechanisms studied by two different test procedures. J Comp Physiol Psychol 48:119–122. 14367584 10.1037/h0041257

[28] Benjamin RM, Akert K (1959) Cortical and thalamic areas involved in taste discrimination in the albino rat. J Comp Neurol 111:231–259. 13798908 10.1002/cne.901110203

[29] Dunn LT, Everitt BJ (1988) Double dissociations of the effects of amygdala and insular cortex lesions on conditioned taste aversion, passive avoidance, and neophobia in the rat using the excitotoxin ibotenic acid. Behav Neurosci 102:3–23. 3281693 10.1037//0735-7044.102.1.3

[30] Hashimoto K, Spector AC (2014) Extensive lesions in the gustatory cortex in the rat do not disrupt the retention of a presurgically conditioned taste aversion and do not impair unconditioned concentration-dependent licking of sucrose and quinine. Chem Senses 39:57–71. 10.1093/chemse/bjt054 24226296 PMC3864164

[31] Pfaffmann C, Norgren R, Grill HJ (1977) Sensory affect and motivation. In: Tonic functions of sensory systems, edited by Wenzel BM and Zeigler HP. Ann.N.Y.Acad.Sci., p. 18–34. 10.1111/j.1749-6632.1977.tb39713.x 276291

[32] Adolphs R, Tranel D, Koenigs M, Damasio AR (2005) Preferring one taste over another without recognizing either. Nature Neuroscience 8: 860–861. 15951808 10.1038/nn1489

[33] Pritchard TC, Macaluso DA, Eslinger PJ (1999) Taste perception in patients with insular cortex lesion. Behav Neurosci 113:663–671. 10495075

[34] Pritchard TC, Norgren R (2004) Gustatory System. In: Paxinos G, Mai J, editors. The Human Nervous System. Boston: Elsevier pp1171–1195.

[35] Small DM, Bernasconi N, Bernasconi A, Sziklas V, Jones-Gotman M (2005) Gustatory agnosia. Neurol 64: 311–317. 10.1212/01.WNL.0000149515.77718.35 15668430

[36] Spector AC (2003) Psychophysical evaluation of taste function in non-human mammals. In: Doty RL, editor. Handbook of Olfaction and Gustation, 2nd Edition New York: Marcel Dekker pp861–879.

[37] Treesukosol Y, Spector AC (2012) Orosensory detection of sucrose, maltose, and glucose is severely impaired in mice lacking T1R2 or T1R3, but Polycose sensitivity remains relatively normal. Am J Physiol Regul Integr Comp Physiol. 303:R218–35. 10.1152/ajpregu.00089.2012 22621968 PMC3404635

[38] Blonde GD, Garcea M, Spector AC (2006) The relative effects of transection of the gustatory branches of the seventh and ninth cranial nerves on NaCl taste detection in rats. Behav Neurosci 120:580–589. 16768610 10.1037/0735-7044.120.3.580

[39] Spector AC, Guagliardo NA, St. John SJ (1996) Amiloride disrupts NaCl vs. KCl discrimination performance: Implications for salt taste coding in the rat. J Neurosci 16:8115–8122. 8987836 10.1523/JNEUROSCI.16-24-08115.1996 PMC6579222

[40] St. John SJ, Markison S, Guagliardo NA, Hackenberg TD, Spector AC(1997) Chorda tympani transection and selective desalivation differentially disrupt two-lever salt discrimination performance in rats. Behav Neurosci 111:450–459. 9106683

[41] Spector AC, Blonde G, Garcea M, Jiang E (2010) Rewiring the gustatory system: specificity between nerve and taste bud field is critical for normal salt discrimination. Brain Res 1310:46–57. 10.1016/j.brainres.2009.11.021 19941834 PMC2812680

[42] Blonde GD, Jiang E, Garcea M, Spector AC (2010) Learning-based recovery from perceptual impairment in salt discrimination after permanently altered peripheral gustatory input. Am J Physiol Regul Integr Comp Physiol 299:R1027–1036. 10.1152/ajpregu.00843.2009 20554935 PMC2957380

[43] Paxinos G, Watson C (2007) The Rat Brain in Stereotaxic Coordinates, Sixth Edition Boston: Elsevier 10.1093/jxb/erm028

[44] Kosar E, Grill HJ, Norgren R (1986b) Gustatory cortex in the rat. II. Thalamocortical projections. Brain Res 379:342–352. 3742226 10.1016/0006-8993(86)90788-2

[45] Katz DB, Simon SA, Nicolelis MA (2001) Dynamic and multimodal responses of gustatory cortical neurons in awake rats. J Neurosci 21: 4478–4489. 11404435 10.1523/JNEUROSCI.21-12-04478.2001 PMC6762775

[46] Fontanini A, Katz DB (2006) State-dependent modulation of time-varying gustatory responses. J Neurophysiol 96:3183–3193. 16928791 10.1152/jn.00804.2006

[47] Stapleton JR, Lavine ML, Wolpert RL, Nicolelis MA, Simon SA (2006) Rapid taste responses in the gustatory cortex during licking. J Neurosci 26:4126–38. 16611830 10.1523/JNEUROSCI.0092-06.2006 PMC6673900

[48] MacDonald CJ, Meck WH, Simon SA (2012) Distinct neural ensembles in the rat gustatory cortex encode salt and water tastes. J Physiol 590:3169–3184. 10.1113/jphysiol.2012.233486 22570382 PMC3406398

[49] Maier JX, Katz DB (2013) Neural dynamics in response to binary taste mixtures. J Neurophysiol 109:2108–17. 10.1152/jn.00917.2012 23365178 PMC3628038

[50] Schafe GE, Bernstein IL (1998) Forebrain contribution to the induction of a brainstem correlate of conditioned taste aversion: II. Insular (gustatory) cortex. Brain Res 800:40–7. 9685579 10.1016/s0006-8993(98)00492-2

[51] Gal-Ben-Ari S, Rosenblum K (2012) Molecular mechanisms underlying memory consolidation of taste information in the cortex. Front Behav Neurosci 5:87 10.3389/fnbeh.2011.00087 22319481 PMC3251832

[52] Spector AC (1995) Gustatory function in the parabrachial nuclei; implications from lesion studies in rats. Rev Neurosci 6:143–175. 8564025 10.1515/revneuro.1995.6.2.143

[53] Moo L, Wityk RJ (1999) Olfactory and taste dysfunction after bilateral middle cerebral artery stroke. J Stroke Cerebr Dis 8:353–354. 17895186 10.1016/s1052-3057(99)80011-1

[54] Cereda C, Ghika J, Maeder P, Bogousslavsky J (2002) Strokes restricted to the insular cortex.Neurology 59:1950–1955. 12499489 10.1212/01.wnl.0000038905.75660.bd

[55] van der Kooy D, Koda LY, McGinty JF, Gerfen CR, Bloom FE (1984) The organization of projections from the cortex, amygdala, and hypothalamus to the nucleus of the solitary tract in rat. J Comp Neurol 224: 1–24. 6715573 10.1002/cne.902240102

[56] Allen GV, Saper CB, Hurley KM, Cechetto DF (1991) Organization of visceral and limbic connections in the insular cortex of the rat. J Comp Neurol 311:1–16. 1719041 10.1002/cne.903110102

[57] Shi CJ, Cassell MD (1998) Cortical, thalamic, and amygdaloid connections of the anterior and posterior insular cortices. J Comp Neurol 399:440–468. 9741477 10.1002/(sici)1096-9861(19981005)399:4<440::aid-cne2>3.0.co;2-1

[58] Lundy RF Jr, Norgren R (2004) Activity in the hypothalamus, amygdala, and cortex generates bilateral and convergent modulation of pontine gustatory neurons. J Neurophysiol 91: 1143–1157. 14627662 10.1152/jn.00840.2003

[59] Kang Y, Lundy RF (2009) Terminal field specificity of forebrain efferent axons to brainstem gustatory nuclei. Brain Res 1248: 76–85. 10.1016/j.brainres.2008.10.075 19028464 PMC2813487

[60] Spector AC, Grill HJ, Norgren R (1993) Concentration-dependent licking of sucrose and sodium chloride in rats with parabrachial gustatory lesions. Physiol Behav 53: 277–283. 8446690 10.1016/0031-9384(93)90205-t

[61] Spector AC, Scalera G, Grill HJ, Norgren R (1995) Gustatory detection thresholds after parabrachial nuclei lesions in rats. Behav Neurosci 109: 939–954. 8554717

[62] Grossman SE, Fontanini A, Wieskopf JS, Katz DB (2008) Learning-related plasticity of temporal coding in simultaneously recorded amygdala-cortical ensembles. J Neurosci 28:2864–2873. 10.1523/JNEUROSCI.4063-07.2008 18337417 PMC6670663

[63] Stone ME, Maffei Am, Fontanini A (2011) Amygdala stimulation evokes time-varying synaptic responses in the gustatory cortex of anesthetized rats. Front Integr Neurosci 5:3 10.3389/fnint.2011.00003 21503144 PMC3071977

[64] Samuelsen CL, Gardner MP, Fontanini A (2012) Effects of cue-triggered expectation on cortical processing of taste. Neuron 74:410–422. 10.1016/j.neuron.2012.02.031 22542192 PMC3340578

